# Combined Eccentric-Isokinetic and Isoinertial Training Leads to Large Ring-Specific Strength Gains in Elite Gymnasts

**DOI:** 10.3390/sports10040049

**Published:** 2022-03-28

**Authors:** Christoph Schärer, Pascal Bucher, Fabian Lüthy, Klaus Hübner

**Affiliations:** Department of Elite Sport, Swiss Federal Institute of Sport Magglingen (SFISM), 2532 Magglingen, Switzerland; bucher.pascal@bluewin.ch (P.B.); fabian.luethy@baspo.admin.ch (F.L.); klaus.huebner@baspo.admin.ch (K.H.)

**Keywords:** artistic gymnastics, strength training, eccentric, rings

## Abstract

In male elite gymnastics, lately, eccentric training is often used to improve the maximum specific strength of static elements on rings. Therefore, in this study, we aimed to investigate the effects of a three-week, gymnastic-specific, eccentric-isokinetic (0.1 m/s) cluster training with a change of stimulus after three of six training sessions (eccentric-isokinetic with additional load) on a computer-controlled training device on the improvement of the elements swallow and support scale on rings. Maximum strength and strength endurance in maintaining the static positions of ten international elite male gymnasts were determined on a weekly basis. After three weeks of training, specific maximum strength and strength endurance increased significantly (strength: swallow: +8.72%, *p* < 0.001; support scale: 8.32%, *p* < 0.0001; strength endurance: swallow: +122.36%; *p* = 0.02; Support Scale: +93.30%; *p* = 0.03). Consequently, top gymnasts can considerably improve ring-specific strength and strength endurance in only three weeks. The separate analysis of the effects of both eccentric-isokinetic training modalities showed that efficiency might even be increased in future training interventions. We suggest using this type of training in phases in which the technical training load is low and monitoring the adaptations in order to compile an individually optimized training after an intervention.

## 1. Introduction

**Figure 1:**
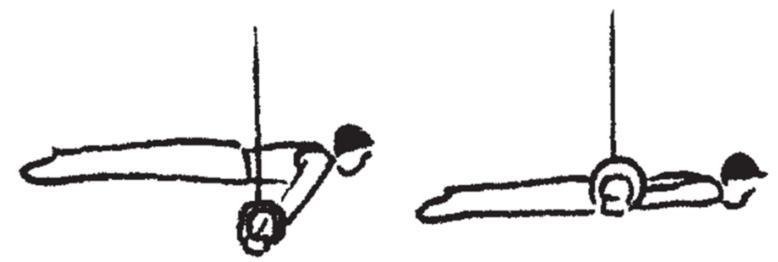
Swallow and support scale. The static elements, swallow (**left**) and support scale (**right**), on rings (FIG, 2022, p. 87) [1].

On rings in men’s artistic gymnastics, a maximum of eight static, eccentric and/or concentric strength elements may be performed within a competition routine. According to its difficulty, each element is assigned an element value from A to H as well as corresponding difficulty values (A: 0.1 points to H: 0.8 points) in the code of points [1]. In addition, all elements are assigned to one of the four element groups (EG I: kip and swing elements; EG II: strength elements and hold elements; EG III: swing to strength hold elements EG IV: dismounts) depending on the movement structure. Strength elements are included in two element groups; therefore, these elements are among the most important to be successful on rings. For a technically clean execution of these elements, a high level of specific maximum strength and strength endurance in the upper extremities is required. In order to overcome gravity and to maintain the static (quasi-isometric) positions of strength elements, muscle work is performed that may be similar to eccentric contractions [2]. In order to achieve a maximal execution score, elements must be held for at least two seconds without angular deviations from the correct position or other executional errors. Angular deviations and other execution errors are classified as small (>0° to <15°), medium (15° to <30°) or large (30° to <45°) and lead to a point deduction by the judges (small: 0.1 points; medium: 0.3 points; large: 0.5 points) [1]. Traditionally, specific concentric exercises with dumbbells or barbells or static strength exercises on rings with additional weight or a counterweight device have been used [3,4] to improve the specific strength and the quality of execution of static elements on rings such as swallow (EG II; difficulty value: D; 0.4 points) and support scale (EG II; difficulty value: C; 0.3 points) (Figure 1).

Recently, eccentric training has become more and more important in athletic training and rehabilitation [5,6], especially where eccentric movements are an integral part of sports activities [7]. As this is the case on rings, where gravity must be overcome while holding static elements, gymnastics coaches have begun using eccentric-isoinertial (constant load) exercises on rings more often to develop the specific strength of the static elements. During eccentric-isoinertial exercises, a supramaximal weight leads to a lengthening contraction of the muscles. In some cases, eccentric-isoinertial training can lead to greater muscle strength gains and muscle hypertrophy as well as greater improvements in muscle coordination compared to concentric strength training [8]. However, the effectiveness for elite athletes is not entirely clear. Compared to concentric contractions, fewer motor units are recruited during the lengthening actions of eccentric-isoinertial exercises, which leads to a higher mechanical stress per motor unit [9]. For this reason, it has been supposed that it is more difficult to control an eccentric movement [5]. On rings, coordinational challenges and the innate instability of the apparatus may provoke excessive torques on the shoulder joint, which may lead to overuse injuries to tendons and muscles. In contrast to eccentric-isoinertial training, eccentric-isokinetic exercises involve maximum voluntary force applied at a constant velocity given by an isokinetic dynamometer with a given range of motion. Both eccentric-isoinertial and eccentric-isokinetic strength training may be used to improve muscle strength in training and rehabilitation [5,10,11]. However, lower level of muscle activation and the unspecific exercises on a dynamometer seem to reduce the effectiveness of eccentric-isokinetic exercises [12]. In order to increase effectiveness of eccentric-isokinetic exercises, a sport-specific range of motion and movement velocity should be applied [13]. Lately, new computer-controlled devices were developed that facilitate to create sport-specific training exercises.

In a previous study, a gymnastics-specific eccentric-isokinetic training exercise in a supine position on a computer-controlled device was developed as a substitute for the coordinationally difficult, specific eccentric-isoinertial exercises on rings with the aims of improving strength while minimizing the risk of injury during training [2]. The intervention consisted of eight training (T) of two to three series with three to four clusters of four repetitions each (T1 and T2: 2 × 4 × 4; T3 and T4: 3 × 3 × 4; T5 and T6: 2 × 3 × 4; T7 and T8: 3 × 4 × 4). While the protocol in that study was successful in both senses, the analysis of training data (recorded by the computer-controlled device) showed that eccentric maximum strength increased in particular from training 2 to training 4 by 9%. Before and after that, the level of eccentric maximum strength stagnated (+2%). In terms of optimizing the training stimulus(what would be in the interest of the coaches and athletes, who are constantly looking for new and even more effective training methods) we concluded that in order to prevent stagnation, future interventions could possibly be shortened and that a more efficient training stimulus could be achieved by changing the configuration after a few training sessions. In this context, a combination of both contraction forms (eccentric-isokinetic training with additional load) could be an interesting new stimulus to boost strength gains when applied in a sport-specific training exercise. However, to the best of our knowledge, eccentric-isokinetic and eccentric-isoinertial training has only been used separately in training interventions. The aim of this combined form of training was that muscles remain under tension throughout the entire cluster sets, thus achieving a higher level of exhaustion and thus providing a new (even greater) eccentric and more complex stimulus to the muscles. For this reason, we hypothesized that the stagnation observed in the previous study would not occur. Moreover, the number of series and sets in training 2 to 4 seemed to be optimal to provoke the desired adaptation. Therefore, only these training configurations should be used in this investigation.

Consequently, the aim of this study was to investigate the effects of a three-week specific eccentric-isokinetic and eccentric-isokinetic training with an additional load on maximum strength and strength endurance of the elements swallow and support scale on rings in elite gymnasts.

## 2. Materials and Methods

### 2.1. Subjects

Ten international and national elite male gymnasts (age: 22.14 ± 2.99 years, height: 167.35 ± 4.07 cm, weight: 63.71 ± 4.04 kg) volunteered to participate in this study. All athletes trained professionally at the national training center and followed during the intervention a similar training program (25 h per week) composed by the head coach of the national team. Gymnasts were informed of benefits and risks prior to participating in the study, which was conducted in accordance with the Declaration of Helsinki, and approved by the Ethics Committee of Bern (Project ID: 2018-00742, 7 June 2018). Informed consent was obtained from all athletes involved in the study. Written informed consent was obtained from the athletes to publish this paper.

### 2.2. Procedure

All gymnasts performed a three-week eccentric training intervention (six sessions (T1–T6), two sessions per week) using the computer-controlled training device 1080 Quantum Syncro (1080 Motion, Lidingö, Sweden). The first three sessions (T1–T3) employed the same eccentric-isokinetic protocol previously used by Schärer, Tacchelli, Gopfert, Gross, Luthy, Taube and Hübner [2]. The second three sessions (T4–T6) adapted the eccentric-isokinetic protocol by applying additional (isoinertial) loads in the form of dumbbells. The dumbbells were used with the intention that the athletes had to keep a continuous muscle tension even between repetitions. The decisions to shorten the overall duration of the intervention and to change the training stimulus after three sessions were based on the results of the previous study, where a plateau in strength gains was observed after only four of eight training sessions. In order to investigate the effects of the eccentric training forms both with and without additional isoinertial loads, gymnasts performed maximum strength and strength endurance tests on a weekly basis (Test 1 (pre-test), Test 2 and 3, Test 4 (post-test)) of the elements swallow and support scale on rings. During the intervention, athletes and coaches were asked not to perform any other ring-specific strength training to avoid biasing the study results.

### 2.3. Maximum Strength and Strength Endurance Tests on Rings

**Figure 2:**
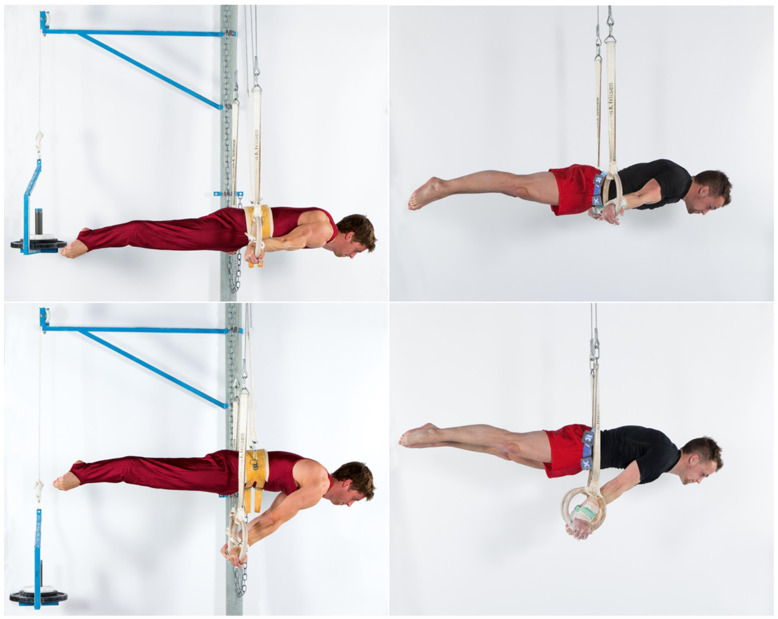
The elements, swallow (**top**) and support scale (**bottom**), were performed with maximum resistance (bodyweight + additional/− counterweight) for five seconds. The image shows the use of the pulley system with counterweight (**left**) and additional weight ((**right**), weight belt).

At each test time point, gymnasts performed a 5 s maximum resistance test of the static strength elements swallow and support scale on rings. The resistance was individually chosen so that gymnasts were able to maintain the elements no longer than 5 s. For this, either a weight belt (additional weight) or a pulley system with a counterweight were used (Figure 2). Athletes were instructed to hold the elements in the best possible position. Attempts were only valid if the required position (joint angle deviation from ideal position <45° according to the Code of Points [1]) could be maintained for the entire 5 s. In order to determine strength endurance of the elements swallow and support scale on rings at test 2, test 3 and post-test, the gymnasts also performed the elements as long as possible, using the maximum resistance determined at pre-test. Between each attempt, gymnasts had at least five minutes of rest in order to recover fully. The attempts were captured with an iPad (iPad Pro 9.7, Apple Corporation, Cupertino, CA, USA). Holding time and holding positions (body angles: swallow—shoulder and body angle; support scale—body and hip angle) were analyzed with the video analysis software Dartfish (Dartfish SA, Fribourg, Switzerland) at the first and the last frame of holding time. Holding time of strength elements started when reaching a stable holding position (with at least two consecutive video frames in the same holding position) and ended when the position was no longer recognized or when the athlete aborted the holding position [2].

### 2.4. Eccentric Training

**Figure 3:**
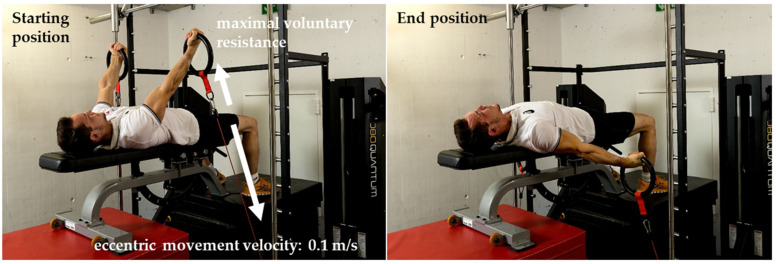
Eccentric isokinetic training with 1080 Quantum. (**Left**): Starting position with arms in the support scale position (45°). (**Right**): End position with arms −15° below the horizontal.

For the eccentric-isokinetic training sessions (T1–T3), gymnasts lay in a supine position with arms in the support scale position (45°) holding a pair of rings. From the start of the eccentric movement (cables were reeled in synchronously with 0.1 m/s), the athlete applied a maximum resistance for approximately 5 s until the arms were in a position of −15° below the horizontal. Thus, the duration of one repetition was comparable to an average holding time of the strength elements during traditional ring training (Figure 3).

For the eccentric-isokinetic training sessions with additional load (T4–T6), the Quantum device operated in the same manner as in T1–T3. The difference was that dumbbells were used in place of rings.

**Figure 4:**
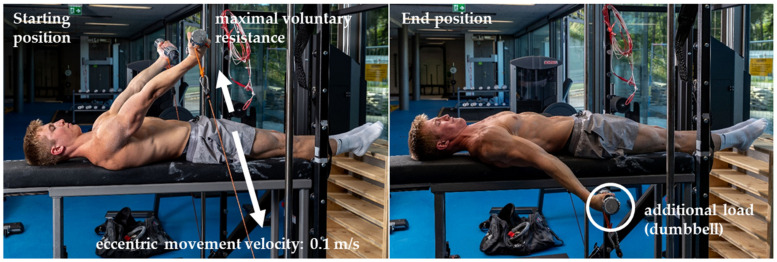
Combined eccentric isokinetic and isoinertial training with the 1080 Quantum Syncro. (**Left**): Starting position with arms in the support scale position. (**Right**): End position with arms −15° below the swallow position.

The goal of the additional weight was to induce a slightly different stimulus after three sessions in order to prevent stagnation of progress. In preliminary tests, an additional load that would provoke greater fatigue but yet still allows gymnasts to complete all sets was determined to be 30% of the minimum applied eccentric force of the eccentric-isokinetic sessions. Therefore, the additional load used in T4–T6 was calculated on an individual basis using the mean of the lowest applied eccentric force from T1 to T3. This resulted in the use of dumbbells between two and six kilograms (1 kg increments) for each hand (mean: 4.38 ± 1.30 kg) (Figure 4).

In order to maintain a high quality (maximum applied force) for each repetition [14], a cluster training method was applied (Table 1). In the first session of each block (T1 and T4), only two cluster sets of 4 × 4 repetitions were performed. In all other sessions, three cluster sets of 3 × 4 were performed. The sets within a cluster were separated by brief rest periods of 20 s. Between the complete cluster sets, in order to recover completely, gymnasts had five minutes of rest.

### 2.5. Statistical Analyses

Descriptive statistics were run on all variables. Normal distribution of the data was confirmed with Shapiro–Wilk test (except for body angles of the static holding position of both elements). In order to calculate overall effects, a one-way analysis of variance (ANOVA) with repeated measures was used. The *t*-tests (post hoc) and effect sizes (Cohen’s d) were calculated to determine differences between the four tests. Effect sizes (d) were classified according to Hopkins, et al. [15] (small: 0.2–0.59, moderate: 0.6–1.19, large: ≥1.2). Overall changes in body angles of the static positions were analyzed using the Friedman-Test. The significance level was set to *p* < 0.05. *p*-values were adjusted using the Holm–Bonferroni correction (Holm, 1979). All statistics were performed with SPSS 22 software (SPSS, Inc., Chicago, IL, USA).

## 3. Results

All participating gymnasts completed the six training sessions. One athlete did not perform post-tests due to illness, and one athlete did not perform the strength endurance tests of Support Scale at test 2 due to shoulder pain unrelated to the studied training intervention. Complete data are presented in Tables S1 and S2.

### 3.1. Maximum Strength

Across the entire three-week intervention, improvements of maximum strength of 8.72% for the element Swallow (*p* < 0.001, η² = 0.65) and 8.32% for support scale (*p* < 0.0001, η² = 0.708) were observed.

For the element Swallow, maximum strength was improved by 5.49% between test 2 and 3 (d = 1.04), while a positive tendency for improvement was found between test 3 and post-test (+2.79%; d = 0.81; *p* = 0.08).

**Figure 5:**
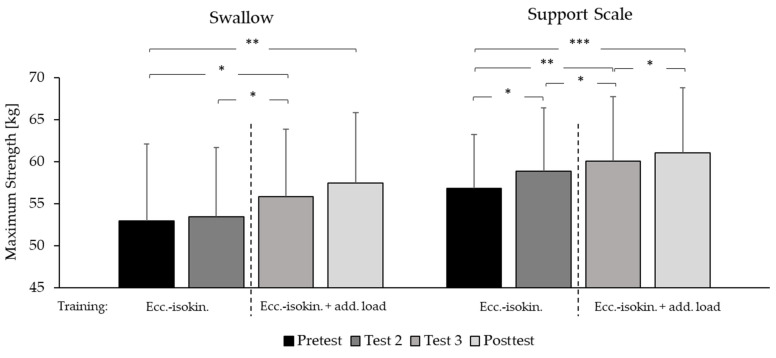
Maximum strength. Mean values and standard deviations for maximal strength (body mass—counterweight or body mass + additional weight) for the swallow and support scale elements when held for 5 s on rings (*n* = 9). Pre-test: before training; Test 2 and 3: after one and two weeks of training, respectively; Post-test: after three weeks of eccentric-isokinetic (ecc-isokin) training. *: significant change: *p* < 0.05; **: significant change: *p* < 0.01; ***: significant change: *p* < 0.001.

Maximum strength performing the element support scale increased by 3.61% between pre-test and test 2 (d = 1.08), by 2.00% between test 2 and 3 (d = 0.88) and by 1.70% between test 3 and post-test (d = 0.98). The average holding time of all maximum strength tests was 4.88 ± 0.29 s for the element swallow and 4.70 ± 0.26 s for support scale (Figure 5). Mean body angles were similar across all maximum strength tests (*p* > 0.05) with slight changes from the start to the end of the holding time (swallow: start (end): shoulder angle: 18.44° ± 1.20° (12.25° ± 1.46°), body angle: 6.62° ± 0.8° (6.73° ± 0.84°); support scale: hip angle: 17.45° ± 2.47° (6.53° ± 1.75°), body angle: 4.85° ± 0.98° (4.78° ± 0.59°)). Overall, the angles corresponded to a maximum deduction of 0.3 points according to the code of points [1].

### 3.2. Strength Endurance

Across the entire intervention, significant improvements in strength endurance for the elements, swallow (*p* = 0.02, η² = 0.46) and support scale (*p* = 0.03, η² = 0.34), were observed.

**Figure 6:**
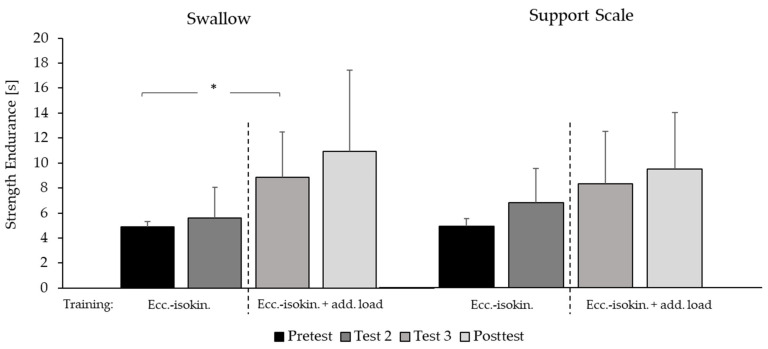
Strength endurance. Mean values and standard deviations for the maximum holding time, with the maximum resistance attained in pre-test (reference) for the swallow and support scale elements on rings (*n* = 9). Pre-test: before training; Test 2 and 3: after one and two weeks of training, respectively; Post-test: after three weeks of eccentric-isokinetic (ecc-isokin) training. *: significant change: *p* < 0.05.

Post-hoc-tests showed a significant improvement of strength endurance for the element swallow between pre-test and test 3 (+80.45%, d = 1.11), and moderate effect sizes between pre- and post-test (+122.36%, d = 0.95; *p* = 0.09) and between test 2 and 3 (+57.62%, d = 0.88, *p* = 0.09) but small effect sizes between pre-test and test 2 (+14.49%, d = 0.34, *p* = 0.34) as well as between test 3 and post-test (+23.23%; d = 0.55, *p* = 0.27). Regarding the improvement of strength endurance of the element support scale, a positive tendency for improvement was found between pre- and post-test (+93.30%, d = 1.08, *p* = 0.06) as well as between pre-test and test 3 (+70.05%, d = 0.89, *p* = 0.08). Between all other tests, moderate effect sizes were observed (<+38.87%, d < 0.73, *p* > 0.177) (Figure 6). The holding positions of both elements were similar across all tests (*p* < 0.05) but changed slightly from the start to the end of the holding time (swallow: start (end): shoulder angle: 18.39° ± 1.20° (10.90° ± 1.95°), body angle: 6.07° ± 2.15° (6.77° ± 1.78°); support scale: hip angle: 8.03° ± 5.66° (6.77° ± 1.76°), body angle: 15.55° ± 8.43° (5.42° ± 1.68°)). Body angles deviated only slightly from the prescribed perfect position, which corresponded to a maximum deduction of 0.3 points in the code of points [1].

## 4. Discussion

This study investigated a novel three-week ring-specific eccentric training cycle similar to a previously published protocol but shorter and with the introduction of additional isoinertial loads halfway through the intervention. Following training, athletes demonstrated significant gains of strength and strength endurance for both static elements on rings.

### 4.1. Maximum Strength

Over the entire three-week intervention, the maximum strength of the elements swallow and support scale improved similarly by upwards of 8%. This improvement was twice as large as in the previous study [2] and occurred after only six instead of eight training sessions. However, improvements for the two elements appeared to occur in somewhat distinct time courses. For the element support scale, strength gain over the three-week intervention was nearly linear, although the greatest weekly increase (> 3%) occurred after the first two eccentric-isokinetic training sessions and weekly improvements decreased slightly thereafter. In contrast, for the element swallow, the largest increases in maximum strength were observed between test 2 and 3 (> 5%) and between test 3 and post-test (> 2%). Therefore, the change in stimulus after the third session appears to have prevented a plateau in support scale strength gains, while the introduction of additional isoinertial loads may have generally been better suited for improving swallow strength. With regard to swallow strength, the question arises as to whether it was the use of additional isoinertial loads or rather the change in stimulus alone that led to greater strength gains in the second and third week of training. A short one- or two-week intervention using only the configuration with additional isoinertial loads could shed some light on this question. If effective, this sort of short training block could be employed with little risk of overtraining and overuse injuries, possibly even shortly before or during the competition season in order to achieve rapid improvements in swallow maximum strength.

In any case, the differing effects of these two different training configurations on the maximum strength of the elements swallow and support scale gives the opportunity to reflect upon the specific adaptation patterns of each.

Adaptations to eccentric exercises are not completely understood, but maximum strength gains from eccentric training appear to occur due to neural, muscle architectural and/or morphological adaptations [16]. Further, it was observed that fast-twitch fibers are selectively activated during lengthening contractions and that IIa- [17,18] and IIx-muscle fibers [18] adapt especially strongly to eccentric training. For this reason, a shift towards a fast phenotype may occur after training [5]. By considering the very short intervention time in this study, it can be assumed that rather functional and/or neuronal adaptations led to the increase in maximum strength. Nevertheless, the eccentric nature of the investigated training protocols, and perhaps, in particular, the configuration with additional isoinertial loads, results in large times under tension and presumably total exhaustion of fast-twitch fibers within each. This may, in general, explain the large strength gains in only three weeks of intervention.

In addition to these factors, the specificity of the movement employed most likely played an important role in eliciting the observed strength adaptations. In the starting position (similar shoulder angle to support scale position), the gymnasts tried to build up a maximal force in a short time and then to maintain maximal resistance through the five-second eccentric movement. During the lowering of the arms, the mechanical strain caused by the eccentric movement increases. Therefore, in the start position (similar shoulder angle to support scale position), muscles are less stretched than in the end position (similar shoulder angle as the swallow position), where the muscle stretch at its maximum and a large mechanical stimulus is acting on the muscular and tendon apparatus. Further, compared to the start position (30° shoulder angle), near the (horizontal) end position, a higher force is applied due to the larger lever arm. This stretch-induced strain may be further intensified by the use of the additional weight. This could explain the larger effects of the training exercise performed with additional weight on the maximum strength performing the element swallow. Contrary to this, in the start position (support scale), the stimulus is more likely to be an explosive movement, with a high rate of force development. With the addition of dumbbells, tension was elevated before the ropes began to pull through the range of motion, which probably diminished the initial rate of force development. Therefore, it might be supposed that specifically in the support scale position, the stimulus was not very different when performing the exercise without or with additional weight. This could explain the slowly decreasing weekly progress of specific maximum strength during the intervention.

### 4.2. Strength Endurance

Overall, strength endurance for both elements, swallow and support scale, increased significantly with large effect sizes from pre- to post-test. Compared to the pre-test, the elements were maintained in the mean between 93% and 133% longer at post-test (with the maximum resistance defined in pre-test).

Regarding the weekly improvements, a similar effect could be observed as for the development of the maximum strength. Strength endurance of the element support scale improved rather continuously, while for the element swallow, a largely greater improvement could be observed when the stimulus was changed with the addition of dumbbells. Therefore, the gains of strength endurance in this study seem to be closely related to the observed maximum strength gains of these two elements on rings. This is contrary to the first study with the same eccentric training device [2], in which the improvements of strength and strength endurance were not so closely related, especially for support scale. Other authors confirm that strength endurance in the upper body is closely related to maximum strength level [19], and therefore, maximum strength is the indispensable prerequisite for strength endurance. However, in the literature, strength endurance and strength levels are usually measured with concentric exercises with rather small coordinative requirements. Further, strength endurance is generally defined as the maximum amount of repetitions with a submaximal weight performed to exhaustion [20]. In this study, strength endurance was specified as the maximum time a quasi-static position on rings could be maintained with a near-maximal resistance. In order to maintain the static positions, a high level of maximal strength, as well as technical and balance skills, are required. A previous study [21] showed that the elements swallow and support scale require different levels of balance skills. For the element support scale, the body’s center of gravity must be maintained above the rings and therefore require more balance skills than to hold the element swallow, where the body’s center of gravity must be maintained at ring level. This requires more maximum strength but involves lower coordination demands.

However, in order to determine strength endurance, the muscle must reach complete exhaustion, which may impair muscular control and balance, maintaining the static position. Muscular fatigue is generally characterized by a loss of muscle force due to exhaustive repetitive movements that result in intramuscular accumulation of metabolites that cause spinal and motor brain structures inhibitions [22]. However, regarding difficult coordinative motor tasks, it was observed that the brain is able to modify the motor control during muscular fatigue, which resulted in a larger variability of muscle activation patterns and probably increases time to exhaustion [23].

Thus, the effects of the two different eccentric training modalities in this study may be summarized as follows. During the “pure” eccentric-isokinetic training form, gymnasts maintain complete control of the intensity by applying more or less force, whereas, during the eccentric-isokinetic training form with the additional weight, there is a minimal force that is required at all times. With the additional weight, greater fatigue can be developed during each set because gymnasts are required to maintain a baseline muscle tension even in a fatigued state. This may have forced the brain to alter motor control and to use different muscle activity patterns in order to prevent the dumbbell from dropping. If so, it seems that gymnasts were able to transfer this ability to alter muscle activation patterns to the technically more difficult element support scale. This could be the main factor that led to the greater increases in strength endurance for both elements compared to the previous study in which only “pure” eccentric-isokinetic training was applied.

The study showed that significant increases in ring-specific maximum strength are possible within a very short time period. It is generally known that muscles adapt much faster to a stimulus than passive structures (tendons, ligaments). Therefore, in the case of a rapid and large increase in strength, an excessive number of supramaximal (also eccentric) loads on the specific muscle groups should be avoided for a few weeks after the intervention in order to allow passive structures to adapt to the new strength level. In order to detect large strength gains, such training interventions must be monitored closely. Finally, this type of training is very strenuous and can therefore limit the quality of sports training. Therefore, eccentric training should be performed in a training phase with a moderate load in technical training.

In individual elite sports, it is difficult to recruit a sufficient number of top-level athletes for scientific studies, and all top-athletes want to benefit from new and promising training methods to preserve their chance of success at an international competition. Therefore, control groups in intervention studies are rare, and it is difficult to verify the effectiveness of a training method in a scientifically correct way. Hence, studies with elite athletes (especially in individual sports) are often limited. However, several athletes who participated in our study won medals at major international events. Consequently, the results in this study can be considered representative, in particular for highly trained top-level athletes.

## 5. Conclusions

In conclusion, this study showed that a three-week specific eccentric-isokinetic training with a change in stimulus after only three training sessions is highly effective for improving the maximum strength and strength endurance of static elements on rings in elite gymnasts. However, the different stimuli used in this study evoked different adaptations for the maximum strength and strength endurance of the elements swallow and support scale. Based on these findings, it is likely that even more efficient interventions could be developed to help gymnasts and coaches optimize ring-specific strength training. Therefore, this study may contribute to the prevention of overload due to inefficient strength training and to a faster improvement of performance if the eccentric training is monitored and applied with caution.

## Figures and Tables

**Table 1:** Eccentric-isokinetic training protocol. Sets, clusters, repetitions (reps), rest duration and time under tension (mean duration per rep ~5 s) for the three-week eccentric-isokinetic training (with additional load) on the computer-controlled training device 1080 Quantum Syncro for the elements swallow and support scale.

Week	Training	Sets/Clusters/Reps (Rest: 5 min/20 s/None)	Time under Tension per Training	Exercise Modality
1	1	2/4/4	~2 min 40 s	Ecc-isokin.
	2	3/3/4	~3 min	Ecc-isokin.
2	3	3/3/4	~3 min	Ecc-isokin.
	4	2/4/4	~2 min 40 s	Ecc-isokin + additional load
3	5	3/3/4	~3 min	Ecc-isokin + additional load
	6	3/3/4	~3 min	Ecc-isokin + additional load

## Data Availability

Raw data can be found in Supplementary Materials.

## Supplementary Materials

The following supporting information can be downloaded at: https://www.mdpi.com/article/10.3390/sports10040049/s1, Table S1. Individual results (resistance: body weight + additional weight/ − counterweight; holding time) at the maximum strength tests of the elements swallow and support scale on rings before (pretest), during (Test 2 and 3) and after (posttest) the three-week eccentric training intervention; Table S2. Individual results (resistance: body weight + additional weight / − counterweight; holding time) at the strength endurance tests of the elements swallow and support scale on rings before (pretest), during (Test 2 and 3) and after (posttest) the three-week eccentric training intervention.

## References

[1] FIG (2022). Code of Points MAG (2022–2024).

[2] Schärer C., Tacchelli L., Gopfert B., Gross M., Luthy F., Taube W., Hübner K. (2019). Specific Eccentric-Isokinetic Cluster Training Improves Static Strength Elements on Rings for Elite Gymnasts. Int. J. Environ. Res. Public Health.

[3] Hübner K., Schärer C. (2015). Relationship between Swallow, Support Scale and Iron Cross on rings and their specific preconditioning strengthening exercises. Sci. Gymnast. J..

[4] Gorosito M.A. (2013). Relative strength requirement for Swallow element proper execution: A predictive test. Sci. Gymnast. J..

[5] Vogt M., Hoppeler H.H. (2014). Eccentric exercise: Mechanisms and effects when used as training regime or training adjunct. J. Appl. Physiol..

[6] Kjaer M., Heinemeier K.M. (2014). Eccentric exercise: Acute and chronic effects on healthy and diseased tendons. J. Appl. Physiol..

[7] Hody S., Croisier J.L., Bury T., Rogister B., Leprince P. (2019). Eccentric Muscle Contractions: Risks and Benefits. Front. Physiol..

[8] Hollander D.B., Kraemer R.R., Kilpatrick M.W., Ramadan Z.G., Reeves G.V., Francois M., Hebert E.P., Tryniecki J.L. (2007). Maximal eccentric and concentric strength discrepancies between young men and women for dynamic resistance exercise. J. Strength Cond. Res..

[9] Douglas J., Pearson S., Ross A., McGuigan M. (2017). Eccentric Exercise: Physiological Characteristics and Acute Responses. Sports Med..

[10] Cowell J.F., Cronin J., Brughelli M. (2012). Eccentric Muscle Actions and How the Strength and Conditioning Specialist Might Use Them for a Variety of Purposes. Strength Cond. J..

[11] Higbie E.J., Cureton K.J., Warren G.L., Prior B.M. (1996). Effects of concentric and eccentric training on muscle strength, cross-sectional area, and neural activation. J. Appl. Physiol..

[12] Guilhem G., Cornu C., Guevel A. (2010). Neuromuscular and muscle-tendon system adaptations to isotonic and isokinetic eccentric exercise. Ann. Phys. Rehabil. Med..

[13] Sakamoto A., Sinclair P.J., Naito H. (2016). Strategies for maximizing power and strength gains in isoinertial resistance training: Implications for competitive athletes. J. Phys. Fit. Sports Med..

[14] Haff G.G., Hobbs R., Sands W.A., Pierce K., Stone M.H. (2008). Cluster Training: A Novel Method for Introducing Training Program Variation. Strength Cond. J..

[15] Hopkins W.G., Marshall S.W., Batterham A.M., Hanin J. (2009). Progressive statistics for studies in sports medicine and exercise science. Med. Sci. Sports Exerc..

[16] Douglas J., Pearson S., Ross A., McGuigan M. (2017). Chronic Adaptations to Eccentric Training: A Systematic Review. Sports Med..

[17] Hortobagyi T., Hill J.P., Houmard J.A., Fraser D.D., Lambert N.J., Israel R.G. (1996). Adaptive responses to muscle lengthening and shortening in humans. J. Appl. Physiol..

[18] Shepstone T.N., Tang J.E., Dallaire S., Schuenke M.D., Staron R.S., Phillips S.M. (2005). Short-term high- vs. low-velocity isokinetic lengthening training results in greater hypertrophy of the elbow flexors in young men. J. Appl. Physiol..

[19] Vaara J.P., Kyrolainen H., Niemi J., Ohrankammen O., Hakkinen A., Kocay S., Hakkinen K. (2012). Associations of maximal strength and muscular endurance test scores with cardiorespiratory fitness and body composition. J. Strength Cond. Res..

[20] Johnson D., Lynch J., Nash K., Cygan J., Mayhew J.L. (2009). Relationship of lat-pull repetitions and pull-ups to maximal lat-pull and pull-up strength in men and women. J. Strength Cond. Res..

[21] Schärer C., Huber S., Bucher P., Capelli C., Hübner K. (2021). Maximum Strength Benchmarks for Difficult Static Elements on Rings in Male Elite Gymnastics. Sports.

[22] Gandevia S.C. (2001). Spinal and supraspinal factors in human muscle fatigue. Physiol. Rev..

[23] Singh T., Latash M.L. (2011). Effects of muscle fatigue on multi-muscle synergies. Exp. Brain Res..

