# Supplementary material for: Combined Eccentric-Isokinetic and Isoinertial Training Leads to Large Ring-Specific Strength Gains in Elite Gymnasts
Source: Sports (Basel). 2022 Mar 28;10(4):49. doi: 10.3390/sports10040049 (PMC9028779; doi:10.3390/sports10040049)
Supplement: Supplementary file 1 [file sports-10-00049-s001.zip › sports-1629580-supplementary.pdf]

Table S1. Individual results (resistance: body weight + additional weight / - counterweight; holding time) at the maximum strength tests of the elements swallow and support scale on rings before (pretest), during (Test 2 and 3) and after (posttest) the three-week eccentric training intervention.

**Maximum strength**																
	**Swallow**								**Support scale**							
**Athlete**	**Pretest**		**Test 2**		**Test 3**		**Posttest**		**Pretest**		**Test 2**		**Test 3**		**Posttest**	
	Resistance (kg)	Time (s)	Resistance (kg)	Time (s)	Resistance (kg)	Time (s)	Resistance (kg)	Time (s)	Resistance (kg)	Time (s)	Resistance (kg)	Time (s)	Resistance (kg)	Time (s)	Resistance (kg)	Time (s)
1	53.70	5.21	55.15	4.70	56.40	4.00	61.60	4.68	48.70	5.04	51.40	5.00	53.90	4.90	56.60	5.11
2	63.70	4.20	59.00	3.60	64.00	4.60	64.50	2.03	63.70	5.64	68.00	6.30	68.00	6.87	68.50	5.11
3	38.70	5.24	41.10	5.86	43.90	3.41	43.70	5.00	46.20	4.83	46.10	4.01	47.65	3.17	48.70	4.80
4	40.10	4.70	40.20	6.00	45.00	3.47	46.00	5.11	50.10	4.97	50.20	3.51	50.00	3.30	52.25	4.30
5	58.50	4.91	59.00	6.04	65.60	5.07	65.20	5.14	61.00	5.21	64.00	6.91	67.60	4.10	67.20	4.44
6	51.70	5.20	54.20	4.63	54.95	4.70	59.00	4.74	61.70	4.27	61.70	4.94	62.95	4.90	64.00	4.31
7	45.60	5.24	46.40	7.28	48.40	5.00	51.90	4.10	54.35	4.41	55.15	4.24	54.65	3.71	59.40	2.75
8	59.00	5.11	58.70	5.10	58.90	5.37	59.30	5.17	61.50	6.04	63.20	3.94	65.40	5.14	67.80	4.94
9	53.60	5.00	55.45	5.38	56.10	5.41	-	-	61.10	4.07	63.70	4.00	65.10	5.51	-	-
10	65.00	4.24	65.40	4.54	65.40	6.14	66.30	5.04	60.00	4.47	65.40	4.41	65.40	5.10	65.30	4.77

Table S2. Individual results (resistance: body weight + additional weight / - counterweight; holding time) at the strength endurance tests of the elements swallow and support scale on rings before (pretest), during (Test 2 and 3) and after (posttest) the three-week eccentric training intervention.

Strength endurance																
	Swallow								Support scale							
Athlete	Pretest		Test 2		Test 3		Posttest		Pretest		Test 2		Test 3		Posttest	
	Resistance (kg)	Time (s)	Resistance (kg)	Time (s)	Resistance (kg)	Time (s)	Resistance (kg)	Time (s)	Resistance (kg)	Time (s)	Resistance (kg)	Time (s)	Resistance (kg)	Time (s)	Resistance (kg)	Time (s)
1	53.70	5.21	53.70	5.70	53.70	10.00	53.70	10.35	48.70	5.04	48.70	8.30	48.70	10.00	48.70	15.66
2	63.70	4.20	63.70	0.00	63.70	4.60	63.70	2.03	63.70	5.64	63.70	11.32	63.70	13.52	63.70	10.35
3	38.70	5.24	38.70	7.01	38.70	10.75	38.70	15.82	46.20	4.83	46.20	4.01	46.20	3.70	46.20	7.88
4	40.10	4.70	40.10	6.00	40.10	17.20	40.10	21.93	50.10	4.97	50.10	3.51	50.10	3.30	50.10	5.40
5	58.50	4.91	-	-	58.50	9.11	58.50	10.35	61.00	5.21	-	-	61.00	9.38	61.00	13.89
6	51.70	5.20	51.70	8.64	51.70	7.15	51.70	17.56	61.70	4.27	61.70	4.94	61.70	5.50	61.70	7.91
7	45.60	5.24	45.60	7.28	45.60	8.04	45.60	10.11	54.35	4.41	54.35	4.24	54.35	3.71	54.35	4.04
8	59.00	5.11	59.00	5.1	59.00	5.37	59.00	5.17	61.50	6.04	61.50	7.20	61.50	14.30	61.50	15.26
9	53.60	5.00	53.60	6.27	53.60	10.15	-	-	61.10	4.07	61.10	8.15	61.10	11.85	-	-
10	65.00	4.24	65.00	4.54	65.00	6.14	65.00	4.84	60.00	4.47	60.00	9.51	60.00	7.98	60.00	4.77
